# Antidotes of Arsenic

**Published:** 1847-11

**Authors:** 


*Antidotes of Arsenic.*—Insoluble salts of arsenic, after remaining a certain time in the stomach, are at last absorbed, and act as violent poisons. The cause of this fact is to be found, according to M. Caventou, in the presence in the stomach of a certain quantity of muriate of ammonia, by the action of which the arsenical preparation becomes soluble. In order to ascertain the different degrees of facility with which the muriate of ammonia dissolves the arsenites of lime, of magnesia, and of iron, M. Caventou instituted experiments, the result of which was to prove that the arsenites of lime was readily dissolved in 115 parts of a saturated solution of muriate of ammonia, the arsenite of magnesia in 330 parts, and the arsenite of iron in 600. Consequently the hydrate of sesquioxide of iron is the antidote which will resist for the longest time the dissolving action of the gastric fluids, and which it will be most proper to exhibit in the first place in cases of poisoning by arsenic.—*Ibid.*

